# FTO m6A Demethylase in Obesity and Cancer: Implications and Underlying Molecular Mechanisms

**DOI:** 10.3390/ijms23073800

**Published:** 2022-03-30

**Authors:** Sarah Kassem Azzam, Habiba Alsafar, Abdulrahim A. Sajini

**Affiliations:** 1 Department of Biomedical Engineering, Khalifa University of Science and Technology, Abu Dhabi P.O. Box 127788, United Arab Emirates; sarah.azzam@ku.ac.ae (S.K.A.); habiba.alsafar@ku.ac.ae (H.A.); 2 Healthcare Engineering Innovation Center (HEIC), Department of Biomedical Engineering, Khalifa University, Abu Dhabi P.O. Box 127788, United Arab Emirates; 3 Center for Biotechnology, Khalifa University of Science and Technology, Abu Dhabi P.O. Box 127788, United Arab Emirates; 4 Department of Genetics and Molecular Biology, College of Medicine and Health Sciences, Khalifa University of Science and Technology, Abu Dhabi P.O. Box 127788, United Arab Emirates; 5 Emirates Bio-Research Center, Ministry of Interior, Abu Dhabi P.O. Box 389, United Arab Emirates

**Keywords:** fat mass and obesity-associated (FTO) protein, N6-methyladenosine (m6A), obesity, adipogenesis, tumorigenesis, cancer

## Abstract

Fat mass and obesity-associated protein (FTO) is the first reported RNA N6-methyladenosine (m6A) demethylase in eukaryotic cells. m6A is considered as the most abundant mRNA internal modification, which modulates several cellular processes including alternative splicing, stability, and expression. Genome-wide association studies (GWAS) identified single-nucleotide polymorphisms (SNPs) within *FTO* to be associated with obesity, as well as cancer including endometrial cancer, breast cancer, pancreatic cancer, and melanoma. Since the initial classification of FTO as an m6A demethylase, various studies started to unravel a connection between FTO’s demethylase activity and the susceptibility to obesity on the molecular level. FTO was found to facilitate adipogenesis, by regulating adipogenic pathways and inducing pre-adipocyte differentiation. FTO has also been investigated in tumorigenesis, where emerging studies suggest m6A and FTO levels are dysregulated in various cancers, including acute myeloid leukemia (AML), glioblastoma, cervical squamous cell carcinoma (CSCC), breast cancer, and melanoma. Here we review the molecular bases of m6A in tumorigenesis and adipogenesis while highlighting the controversial role of FTO in obesity. We provide recent findings confirming FTO’s causative link to obesity and discuss novel approaches using RNA demethylase inhibitors as targeted oncotherapies. Our review aims to confirm m6A demethylation as a risk factor in obesity and provoke new research in FTO and human disorders.

## 1. Introduction

The fat mass and obesity-associated (*FTO*) gene was the first gene to show the strongest association with polygenic obesity. The FTO protein encoded by the *FTO* gene belongs to a family of dioxygenases that depend on Fe (II) and 2-oxoglutarate (2OG) for their enzymatic activity [1]. Enzymatically, FTO is an RNA N6-methyladenosine (m6A) demethylase in eukaryotic cells [2]. m6A is the most abundant internal post-transcriptional modification in messenger RNA (mRNA) with high conservation across human and mouse [3]. FTO was later identified to also demethylate *N*^6^,2′-*O*-dimethyladenosine (m6A_m_)-dimethyl-modified mRNAs and reduce their chemical stability [4]. m6A is a highly-dynamic and reversible modification with writers depositing them on RNA while erasers remove them [5,6]. Once m6A is deposited on RNA, different groups of proteins, called readers, facilitate downstream functions. Several cellular processes are modulated via m6A including alternative splicing, mRNA stability, translation, transportation, degradation, and gene regulation [3,7,8,9,10,11]. Additionally, the co-localization of FTO and components of m6A methylation complex with splicing proteins in nuclear speckles, which are involved in the assembly of mRNA-processing factors, further supports regulatory roles of m6A in RNA processing [2,12]. The imbalance of m6A has been associated with various human disorders, such as obesity, diabetes, heart failure, brain diseases, and various cancers [13,14,15,16,17,18]. In GWAS, FTO constantly scored high among obese and cancer cohorts. Molecularly, variation in *FTO* could explain the m6A perturbation found in both obesity and cancer. Hence, many studies have explored the molecular mechanisms of FTO in adipogenesis and tumorigenesis with conflicting results. The inconsistency arises from the fact that most FTO single-nucleotide polymorphisms (SNPs) associated with overweight are intronic. This has divided scientists studying FTO in obesity into groups hypothesizing that FTO introns act as cis-regulatory sites for adjacent genes, while other groups hypothesize that FTO introns act as auto-regulators. Here we review studies from both groups and support the fact that FTO does directly modulate obesity on the m6A level. We also discuss FTO’s mechanistic role in cancer and shed some light on m6A demethylase inhibitors as novel cancer-targeted therapies. We hope our review encourages aligned views in studies involving FTO, obesity, and cancer.

## 2. FTO Association with Obesity as an m6A Demethylase (Obesogenic Role)

### 2.1. FTO Association with Obesity in Genome-Wide Association Studies and Animal Models

*FTO* was the first gene having the strongest genetic association with polygenic obesity. Using GWAS, scientists identified significant SNPs in the first intron of *FTO* that were associated with obesity. These initial screens were done in 2007 among European populations [19,20,21], and later confirmed in other populations, including Hispanics [22,23], East Asians [24,25,26], Native Americans [27], South Asians [28,29], Africans [30] and Middle-Easterns [31,32,33,34,35,36]. Genomically, intron 1 and 2 of the *FTO* gene show the strongest genome- association with obesity, where 89 variants were identified [19,21]. Phenotypically, the higher body-mass index (BMI) recorded in individuals carrying *FTO* risk variants may be attributed to increased energy intake [37,38,39] and reduced food satiety [40], yet not due to impaired energy expenditure [37,41]. Given the obstacles of exploring FTO in humans, researchers used mouse models to delineate *FTO’s* roles in energy homeostasis and fat-mass regulation [13,42,43,44,45,46,47]. In mice, lower fat mass and body weight were reported when *FTO* was knocked out or ablated by loss-of-function mutations [42,43,44,45]. Conversely, *FTO* overexpression led to higher body and fat mass, as seen from knock-in mice models as well as plants’ increased yield and biomass, upon *FTO* overexpression [13,48]. Apart from weight, mice lacking *FTO* also suffered from postnatal growth retardation, which highlights FTO’s role in the central nervous system development [42,44]. In pigs, *FTO* knockdowns reduced lipid accumulation supporting FTO’s role in adipogenesis [49]. Humans homozygous for *FTO* mutations that deactivate it enzymatically, suffer from multiple congenital abnormalities, severe growth retardation and die in infancy, highlighting an essential role of *FTO* in normal human development [50,51]. Interestingly, none of the heterozygous parents of the aforementioned *FTO*-enzymatic mutant were clinically obese, suggesting a single functional copy of FTO was sufficient to minimize the risk of obesity [50]. However, further studies investigating the relation between FTO heterozygosity and obesity are needed. All in all, data across different species support FTO’s involvement in higher body mass.

### 2.2. FTO Molecular Association with Obesity as an m6A Demethylase

GWAS represent a powerful tool in establishing connections between FTO variants and phenotypical features of obesity such as BMI and waist circumference. Nevertheless, translating the findings from such studies to molecular roles of FTO variants in obesity is a continuous challenge. Since the initial identification of FTO as an m6A demethylase, a number of recent studies began to unravel a connection between FTO-dependent m6A demethylation and obesity. Karra et al. uncovered a novel role of FTO as a potential regulator of ghrelin, the hunger hormone, by comparing humans homozygous for *FTO* SNP rs9939609 risk allele, with wild-type carriers [52]. They found that blood cells carrying the risk allele expressed elevated *FTO* mRNA levels which in turn led to higher ghrelin expression [52]. This study highlighted a potential mechanism underlying the obesity-prone eating behavior observed in *FTO* rs9939609 individuals. However, further experiments are required to confirm whether modulating ghrelin is sufficient to explain FTO’s molecular role in obesity.

Using porcine adipocytes, m6A levels were shown to negatively regulate lipid accumulation [49]. The inverse regulatory effect of m6A was confirmed via *FTO* overexpression and knockdown studies, as well as by exogenously regulating m6A levels using cycloleucine, a chemical inhibitor of RNA methylation [49,53]. A study by Zhao et al. [10] using mice 3T3-L1 pre-adipocytes reported an inverse correlation during adipogenesis between m6A levels and *FTO* expression. Specifically, m6A demethylation by FTO weakens the binding affinity of the splicing regulatory (SR) protein SRSF2. The Runt-related transcription factor 1 (RUNX1T1), an adipogenic gene, is a target of SRSF2, and has two splice variants; a short (S) one and a long (L) one.

The perturbed binding of SRSF2 results in the skipping of exon 6 and the generation of the RUNX1T1 S variant, which induces pre-adipocyte differentiation. In contrast, *FTO* knockdown leads to m6A-accumulation and recruitment of SRSF2 towards RUNX1T1 transcripts. SRSF2 assists in generating the RUNX1T1 L variant by including exon 6 [10]. This example illustrates how FTO demethylation of m6A regulates RNA processing and pre-adipocyte differentiation via alternative splicing. Another study by Zhang et al. [54] also highlighted the role of FTO demethylation in adipocytes differentiation by using murine 3T3-L1 pre-adipocytes. They showed that *FTO* knockdown perturbed 3T3-L1 pre-adipocytes differentiation into mature adipocytes. Conversely, *FTO* overexpression in 3T3-L1 pre-adipocytes increased adipogenesis by lowering m6A levels. Interestingly, the expression of an *FTO* missense mutant, lacking its catalytic activity (R96Q), impeded differentiation but had no effect on m6A levels; potentially explained via competing with endogenous FTO for substrate binding [54]. The mutation of Arg96 to Gln in the R96Q *FTO* mutant compromises its demethylase activity; given the essential role of Arg96 as an interacting residue in substrate binding [55]. The use of Rosiglitazone, a PPARγ agonist, in R96Q mutant expressing cells was shown to partially overcome adipogenesis inhibition; suggesting that FTO plays a role in adipogenesis at least partially via PPARγ, a master adipogenic regulator [54].

In-vitro studies connecting FTO to adipogenesis were further confirmed by Merkestein et al. [46]. Their study provided mechanistic insights relating *FTO* upregulation with obesity; where it regulates early adipogenesis during the mitotic clonal expansion (MCE) phase. In line with a previous study [10], FTO is reported to regulate the splicing of the adipogenesis-related transcription factor RUNX1T1. Elevated pro-adipogenic RUNX1T1 S-isoform stimulates the MCE phase in adipocytes. Stimulating the MCE phase, through FTO, enhances adipogenic differentiation via increasing D-type Cyclin genes (Cyclin D1 and Cyclin D3) and PPARγ and C/EBPα, master regulators of adipogenesis. Merkestein et al. showed that *FTO* knockdown after MCE (48 h post adipogenic induction) did not influence adipogenic genes; highlighting the time-sensitive role of FTO in adipogenesis. In-vivo studies of mice linked the role of high-fat diet (HFD) in *FTO*-overexpressing mice (FTO-4), as fat pads from such mice displayed increased adipocytes number, indicating hyperplasia induction upon HFD [46].

Recent studies showed conflicting results in regards to the effect of *FTO* SNPs on *FTO* expression and obesity development. Some studies indicate that FTO SNPs work by modulating adjacent genes such as *IRX3* and *RPGRIP1L* [56,57]. Other studies show that *FTO* SNPs increased *FTO* expression, hence act as autoregulators [52,58]. The study by Merkestein et al. supported the role of *FTO* in adipogenesis independent of either *IRX3* or *RPGRIP1L* [46]; implying that gain-of-function variants in *FTO* may stimulate adipogenesis in carriers of *FTO* variant on an HFD.

The involvement of FTO in energy expenditure regulation was pointed on a molecular level, via the browning effect of white adipose tissue in mice and humans [59,60]. Tews et al. demonstrated that *FTO* deficiency results in the upregulation of uncoupling protein 1 (UCP-1) in adipocytes; hence, enhancing mitochondrial uncoupling and energy expenditure in brown adipocytes [59]. This raises the speculation that FTO may increase body weight via reducing UCP-1 expression and inhibiting the browning of white adipose tissue. Claussnitzer group [60] studied the mechanism of *FTO* variant (T-to-C) rs1421085, in connection with obesity; as it was shown to disrupt a conserved repressor motif (ARID5B). The loss of the later motif causes an upregulation of downstream targets *IRX3* and *IRX5* during early adipocyte differentiation. Consequently, a reduction in heat dissipation and loss of mitochondrial thermogenesis occurs (a process which is regulated via UCP1, peroxisome proliferator-activated receptor γ coactivator 1 α (PGC1α), and PR-domain containing 16 (PRDM16)). This shift from energy-dissipating beige adipocytes into energy-storing white adipocytes results in an increased lipid storage capacity and weight gain [60]. Studies linking *FTO* expression with white adipose tissue browning represent novel avenues for better obesity management in the future [59,60].

FTO activity as an m6A demethylase is also correlated with the regulation of lipid accumulation in skeletal muscle cells via its regulatory role in the AMP-activated protein kinase (AMPK) signaling pathway [61]. The energy sensor AMPK negatively regulates lipid accumulation in skeletal muscle cells, at least partially via regulating *FTO* expression levels and FTO-dependent m6A demethylation. Active AMPK mitigates *FTO* expression, resulting in higher m6A methylation levels (positive regulation) in skeletal muscles which reduces lipid accumulation (negative regulation). Wu and colleagues [61] showed that m6A modifications increase lipid accumulation via decreasing lipolysis and oxidation-associated transcripts (adipose triglyceride lipase (*ATGL)*, hormone-sensitive lipase (*HSL*), and *PGC1 α*) while stabilizing lipid synthase related transcripts (CCAAT/enhancer binding protein α *(C/EBPα)* and fatty acid synthase (*FAS*)). These regulatory mechanisms could act as manipulation tools for lipid deposition in skeletal muscle by targeting AMPK and/or utilizing m6A-associated drugs [61].

Moreover, FTO seems to play a regulatory role in cell cycle progression by modulating m6A and its reader YTHDF2 as reported by Wu et al. [62]. FTO was initially reported to regulate early adipogenesis by regulating MCE, a stage occurring within 48 h of adipogenic induction [46]. In 2018, Wu et al. provided further mechanistic details, whereby *FTO* knockdown resulted in higher m6A levels on *cyclin A2* mRNA (*CCNA2*) and *cyclin-dependent kinase 2* mRNA (*CDK2*). CCNA2 and CDK2 are two key regulators of early mitotic events, where their cell cycle role is focused on mediating the S to G2 phase transition [63]. The accumulation of m6A on both *CCNA2* and *CDK2* mRNAs signals degradation via YTHDF2; thus impairing cell cycle progression and suppressing adipogenesis in 3T3-L1 pre-adipocytes [62]. As an m6A reader, YTHDF2 was previously reported to regulate mRNA degradation [7]. It would be interesting to explore the roles of other m6A- readers in adipogenesis such as YTHDF1 and YTHDF3, given their functions in translation [8,64].

Recently, the Zinc finger protein 217 (Zfp217) was uncovered as another player in FTO-dependent adipogenic regulation [65]. Zfp217 is an oncogenic protein overexpressed in multiple human cancers [66,67,68]. Transcriptionally, Zfp217 binds to *FTO’s* promoter region and increases its expression; thereby reducing global m6A levels. *Zfp217* ablation in 3T3-L1 pre-adipocytes halts adipogenic differentiation by decreasing the expression of key adipogenic genes namely *PPAR*γ and *aP2*. In addition to its transcriptional activation role of *FTO*, Zfp217 interacts with YTHDF2 to modulate mRNA m6A modifications. Specifically, Zfp217 sequesters YTHDF2 and shifts the equilibrium in YTHDF2-FTO competition over m6A sites, by favoring FTO m6A demethylation activity. This study by Song et al. supports previous findings linking *Zfp217* overexpression with adipogenesis; whereby Zfp217 promotes adipogenesis via transcriptional and post-transcriptional mechanisms [65,69].

Another FTO-dependent factor in adipogenesis is nicotinamide adenine dinucleotide phosphate (NADP); highlighting a direct association between metabolism and RNA m6A demethylation [70]. In 3T3-L1 pre-adipocytes NADP directly binds to FTO and enhances its demethylase activity; hence promoting m6A removal and adipogenesis. In this study, both control and *FTO* knockdown 3T3-L1 pre-adipocytes were treated with NADPH prior to adipogenic induction; and notably, NADPH enhanced adipogenesis in control pre-adipocytes. However, *FTO* knockdown impaired NADPH’s ability to enhance adipogenesis, as shown from lipid staining and triglyceride accumulation. In line with 3T3-L1 pre-adipocytes results, Wang et al. also demonstrated that NADP modulated m6A levels in *FTO*-knockout mice [70]. It will be interesting to explore how NADP enhances FTO and whether this process is found in human adipocytes.

Novel mechanistic insights were recently discovered by Wang et al. [71] which connects m6A modifications with adipogenesis and autophagy. Specifically, *FTO* knockdown in 3T3-L1 pre-adipocytes and in porcine primary pre-adipocytes resulted in higher m6A levels on two autophagy-related genes *ATG5* and *ATG7;* hence marking them for degradation by YTHDF2. Low ATG5 and ATG7 proteins lead to autophagy and adipogenesis inhibition. Pinpointing ATG5 and ATG7 as key regulators in FTO-dependent autophagy and adipogenesis further contributes to our understanding of how FTO could be regulating obesity. Recently, Wu et al. unveiled how FTO can promote thermogenesis and white-to-beige adipocyte transition. Mechanistically, FTO loss in adipose tissue increases m6A accumulation on hypoxia inducible factor 1 subunit alpha (*HIF1A)* mRNAs. Abundant m6A enhances HIF1A translation by m6A-reader YTHDC2. Transcriptionally, HIF1A activates thermogenic genes such as *PGC1A*, *PRDM16*, and *PPARG*, thereby promoting a “browning” process of white adipose tissue [72].

**Figure 1:**
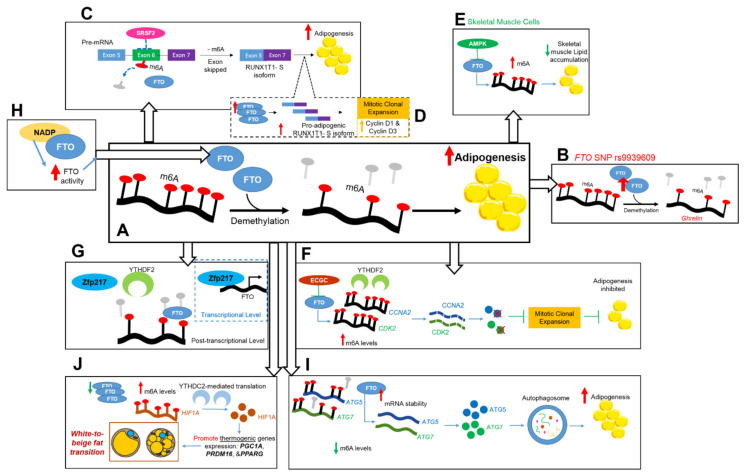
FTO molecular mechanisms in association with adipogenic pathways. (**A**) Inverse correlation between FTO overexpression and lipid accumulation as reported in porcine adipocytes. (**B**) Overexpression of FTO in human subjects homozygous for *FTO* SNP rs9939609 risk allele, resulting in increased levels of the hunger hormone ghrelin. (**C**) m6A and alternative splicing regulation: perturbed binding of SRSF2 to RUNX1T1 results in the skipping exon 6 and generating the pro-adipogenic RUNX1T1 S variant. (**D**) Mechanistically, RUNX1T1 S-isoform promotes adipogenic differentiation via increasing D-type Cyclin genes (Cyclin D1 & D3) during the MCE phase. (**E**) FTO regulatory role in skeletal muscle cells’ lipid accumulation capacity: AMPK activation downregulates FTO, resulting in reduced lipid accumulation. (**F**) FTO regulatory role in adipocyte cell cycle progression: FTO inhibition results in m6A hyper-methylation of two mitotic regulators’ transcripts CCNA2 and CDK2, which are recognized and degraded by YTHDF2 m6A reader; impairing cell cycle and suppressing adipogenesis. (**G**) Zfp217 interaction with FTO promotes adipogenesis via transcriptional and post-transcriptional regulatory mechanisms. (**H**) Association between metabolism and RNA m6A demethylation: NADP binding to FTO enhances its activity and promotes adipogenesis. (**I**) Connecting m6A role in adipogenesis with autophagy: FTO reduces m6A levels on two autophagy-related genes ATG5 and ATG7, stabilizing them from decay by YTHDF2; thus promoting autophagy and adipogenesis. (**J**) FTO promotes thermogenesis and white-to-beige fat transition: FTO knockdown produces m6A hyper-methylated HIF1A and increases its translation through a YTHDC2-mediated process; HIF1A, in turn, activates thermogenic genes PGC1A, PRDM16, and PPARG, which promote white adipocyte “browning”, as an anti-obesity approach. Abbreviations: RUNX1T1: Runt-related transcription factor 1; SRSF2: splicing regulatory protein; MCE: mitotic clonal expansion phase; AMPK: AMP-activated protein kinase; CCNA2: cyclin A2; CDK2: cyclin-dependent kinase 2; Zfp217: Zinc finger protein 217; NADP: nicotinamide adenine dinucleotide phosphate; ATG5: autophagy-related gene 5; ATG7: autophagy-related gene 7; HIF1A: hypoxia inducible factor 1 subunit alpha.

In Table 1 below, we summarize a list of studies classifying FTO as an obesogenic regulator and provide an illustrative summary of those studies in Figure 1.

## 3. FTO m6A Demethylase Association with Various Cancers

### 3.1. FTO Is Overexpressed in a Number of Human Cancers

Further to its association with obesity, epidemiological studies link *FTO* SNPs with higher risks of endometrial cancer [74,75,76], breast cancer [77,78], pancreatic cancer [76,79,80] and melanoma [81]; with some variations among different races. Some of *FTO* SNPs were reported to be BMI-related [75,77,79] while others were independent of BMI [74,78,80,81]. These contradicting results contribute further to the complexity of the re-lationship between obesity and cancer. Emerging studies observe that m6A modifications and their modifying enzymes are dysregulated in various cancers, specifically acute myeloid leukemia (AML), glioblastoma, cervical squamous cell carcinoma (CSCC), breast cancer and melanoma. These observations support FTO and m6A modifications in tu-morigenesis, hence making FTO a potential target for precision medicine in cancer, as shall be discuss herein.

### 3.2. Preliminary Mechanistic Investigations of FTO Oncogenic Role

The connection between endometrial cancer and *FTO* variants [74,75] were first reported in 2011 by Zhang et al. [82]. In particular, *FTO* expression was constantly abundant in both endometrial cancer specimens and endometrial cancer cell lines. It was later discovered that β-estradiol (E2) drove *FTO* overexpression by binding to estrogen receptor (ER), which then modulated FTO by activating PI3K/AKT and MAPK pathways. This in turn upregulates cyclin D1 and metalloproteinases MMP2/9 [82]. Interestingly, the association between FTO and endometrial carcinoma did not consider FTO as an m6A demethylase, since FTOs demethylation activity was not reported until later that same year [2]. In breast cancer *FTO* is also overexpressed in mastectomy tissue; specifically in HER-2 high subtypes [83]. Clinically, *FTO* abundance is associated with poor prognosis and early relapse of endometrial cancer [84]. Additionally, estrogen activates mTOR signaling [84], a pathway implicated in endometrial cancer development and progression [85]. Thus, leading to FTO nuclear localization, and increasing endometrial cancer proliferation [84]. 

The oncogenic role of FTO in breast cancer was studied by comparing breast cancer cell lines to normal breast cells. This comparison revealed that *FTO* was overexpressed in breast cancer cells; denoting it as a potential diagnostic marker in breast cancer. Mechanistically, *FTO* overexpression in breast cancer cells increases energy metabolism via the PI3K/AKT signaling pathway, which is abnormally activated in many human cancers [86,87,88]. *FTO* overexpression upregulates pyruvate kinase and hexokinase, and increases ATP generation; thus, promoting aerobic glycolysis [86]. The FTO oncogenic role was also reported in human gastric cancer (GC), where FTO was upregulated on both protein and mRNA levels. Similar to breast cancer FTO abundance is associated in GC with metastasis; marking it as a poor prognostic marker [89].

### 3.3. The Oncogenic Role of FTO as an m6A Demethylase and Associations with Cancers

The mechanistic roles of FTO in tumorigenesis are currently being elucidated by various research groups in terms of its m6A demethylase activity.

A study by Li et al. [90] discovered a previously unrecognized oncogenic role of FTO in acute myeloid leukemia (AML). They showed that *FTO* was abnormally regulated in subtypes of AML, specifically t(11q23)/*MLL*-rearranged, t(15;17)/*PML-RARA*, *FLT3*-ITD, and/or *NPM1*-mutated AMLs. *FTO* expression enhanced AML cellular viability and proliferation while inhibiting apoptosis as well as significantly promoting Leukemogenesis in-vivo [90]. Furthermore, depletion of *FTO* significantly enhanced all-trans-retinoic acid (ATRA)-induced AML cell differentiation. Here, FTO regulates the mRNA stability of two genes: Ankyrin repeat and SOCS box protein 2 (*ASB2)* and retinoic acid receptor α (*RARA)* by controlling m6A levels. Interestingly, it was demonstrated that FTO’s oncogenic role was independent of YTHDF2 and YTHDF1; indicating that an alternative m6A reading process might be involved [90]. Cui et al. established a causative link between m6A modifications and glioblastoma, a highly aggressive form of brain cancer [16]. They show that the use of FTO inhibitor (Meclofenamic acid MA) [91] to target glioblastoma stem cells (GSCs), results in suppressing GSC-induced tumorigenesis as well as increasing the lifespan of GSC-engrafted mice [16]. These findings suggested that m6A erasers, like FTO, represent interesting targets for glioblastoma therapy. Su et al. [92] report the use of R-2-hydroxyglutarate (R-2HG), a previously reported oncometabolite [93,94], as a small-molecule inhibitor of FTO; displaying anti-proliferative effects in leukemia and glioma. In fact, R-2HG exerts its anti-tumor effects via inhibiting FTO enzymatic activity [92]. This in turn increases m6A levels on FTO oncogenic mRNA targets, specifically reported in this study *MYC/CEBPA*; hence reducing their stability and suppressing relevant oncogenic pathways. Moreover, Su et al. reported that R-2HG administration resulted in synergistic interactions and displayed better therapeutic effects when combined with first-line chemotherapy drugs such as daunorubicin or decitabine, as verified in-vivo using leukemia mice models xeno-transplanted with R-2HG sensitive leukemic cells [92].

**Figure 2:**
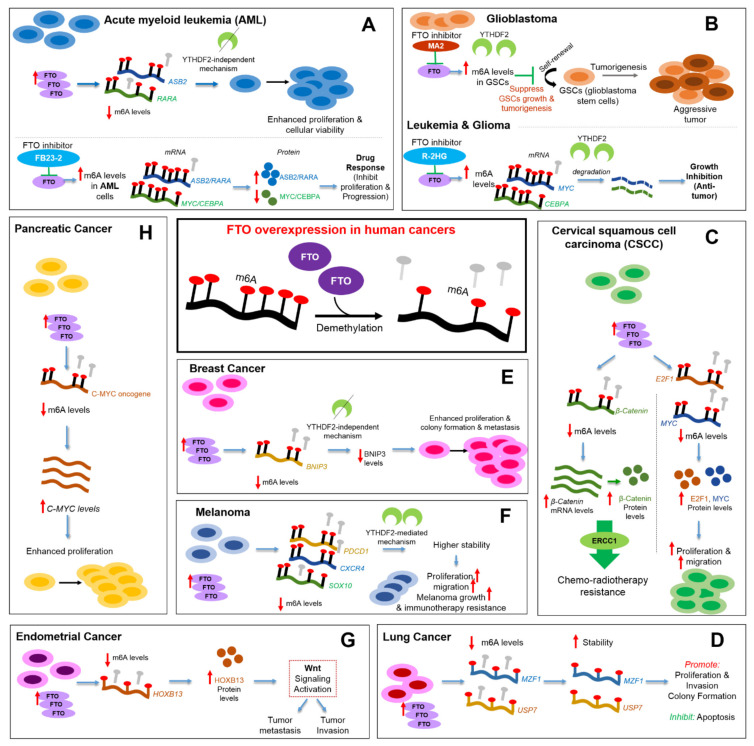
FTO upregulation in various human cancers and its molecular implications as an m6A demethylase, indicated by the regulation of different targets and affected signaling pathways. (**A**) FTO upregulation in AML modulates ASB2/RARA levels, hence, promoting AML cellular viability and proliferation. FTO inhibition via FB23-2 inhibits AML progression, by upregulating ASB2/RARA and downregulating MYC/CEBPA. (**B**) Given FTO’s tumorigenic role in glioblastoma, its inhibition via MA2 suppresses GSCs self-renewal and tumorigenesis. Additionally, inhibiting FTO using R-2HG exerts anti-tumor functions in lethal glioma and leukemia by modulating MYC/CEBPA levels. (**C**) FTO upregulation in CSCC induces chemo-radiotherapy resistance by increasing β-catenin and activating the ERCC1 pathway. E2F1/MYC are also upregulated targets in CSCC that promote oncogenic functions. (**D**) FTO overexpression in lung cancer upregulates MZF1 and USP7 levels via modulating their m6A levels. Thus, promoting lung cancer cells’ proliferation, invasion, and colony formation, while inhibiting their apoptosis. (**E**) In breast cancer cells, FTO upregulation demethylates BNIP3 as a downstream target and downregulates it in a YTHDF2-independent manner, and promoting oncogenic roles like metastasis. (**F**) In melanoma, upregulated FTO and reduced m6A levels, stabilize melanoma-promoting genes namely, PDCD1, CXCR4, and SOX10 in a YTHDF2-mediated process. Consequently, increasing melanoma migration, proliferation, and immunotherapy resistance. (**G**) In endometrial carcinoma, higher HOXB13 expression activates Wnt signaling and promotes metastasis. (**H**) In pancreatic cancer, FTO overexpression leads to upregulating c-MYC oncogene and enhancing pancreatic cancer proliferation. FTO’s additional role as a tumor suppressor in pancreatic cancer is discussed in the text. Abbreviations: ASB2: Ankyrin repeat and SOCS box protein 2; RARA: retinoic acid receptor α; GSCs: glioblastoma stem cells; MA: Meclofenamic acid; R-2HG: R-2-hydroxyglutarate; ERCC1: excision repair cross-complementation group 1; MZF1: Myeloid Zinc Finger Protein 1; USP7: ubiquitin-specific protease-7; BNIP3: BCL2 Interacting Protein 3; HOXB13: homeobox transcription factor.

Zhou et al. study [95] was the first to report FTO’s role in treatment response of a solid tumor, specifically cervical squamous cell carcinoma (CSCC). In their study, they suggest that FTO/β-catenin/ERCC1 axis plays a critical role in regulating chemo-radiotherapy resistance in CSCC. *FTO* mRNA levels were upregulated in CSCC tissues relative to adjacent normal tissues. FTO promoted chemo-radiotherapy resistance in-vitro and in-vivo, via demethylating *β-catenin* transcripts; thus upregulating its mRNA and protein levels. Subsequently, excision repair cross-complementation group 1 (ERCC1) was found to be an activated downstream target contributing to the FTO/β-catenin induced chemo-radiotherapy resistance in CSCC cells (Figure 2) [95]. FTO serves as an oncogenic regulator, by modulating m6A levels on *MYC* and *E2F1*; thus increasing cervical cancer’s migration and proliferation [96].

FTO has a pro-tumorigenic role in lung cancer, as evident from its overexpression in lung cancer tissues and cell lines [97,98]. FTO wildtype overexpression, but not its mutant enzymatically-inactive forms, promoted oncogenic functions in lung cancer cells, including proliferation, invasion, and colony formation ability. Mechanistic analyses revealed two functional targets of FTO; first, upregulation of Myeloid Zinc Finger Protein 1 (MZF1) in lung squamous cell carcinoma (LUSC), and second: ubiquitin-specific protease-7 (USP7) in non-small cell lung cancer (NSCLC). Both targets’ stability was dependent on FTO m6A demethylase activity. FTO upregulation in lung cancer cells and the resulting reduced m6A levels on *MZF1* and *USP7* increased their stability; thereby, promoting oncogenic functions [97,98].In colon cancer cell lines, Tsuruta et al. reported the upregulation of FTO and its target: Programmed death 1 ligand (PD-L1) [99]. PD-L1 is one of the immune-check point molecules, which when highly expressed on tumor cells permits their escape from immune surveillance [100].

*FTO* was also upregulated in human breast cancer [101]. Niu et al. demonstrated that FTO promoted breast cancer cell proliferation, colony formation, and metastasis. Mechanistically, FTO exerted its oncogenic role in breast cancer via demethylating BCL2 Interacting Protein 3 (BNIP3) [101], a pro-apoptosis member in the Bcl-2 apoptotic protein family [102]. Consequently, downregulating BNIP3 and resulting in reduced apoptosis of breast cancer cells. FTO demethylase action on BNIP3 negatively regulated its expression and modulated its stability, through a YTHDF2-independent manner [101]. This is in line with an earlier study which reported the regulation of AML associated oncogenic target genes *ASB2* and *RARA* by FTO via a YTHDF2-independent mechanism [90]. This indicates that an alternative reading process of m6A-modified transcripts and thus alternative stabilizing exists and ought to be explored.

A pro-tumorigenic role of FTO was recently discussed in melanoma, illustrating the upregulated *FTO* levels in human melanoma samples and multiple melanoma cell lines, and reporting that FTO promotes melanoma tumor growth in-vivo [103]. Molecular details were discussed by Yang et al. [103], in which *FTO* knockdown resulted in m6A hyper-methylated melanoma-promoting genes namely, *PD-1 (PDCD1)*, *CXCR4,* and *SOX10*; consequently reducing their mRNA stability, at least partially via a YTHDF2-mediated mechanism. The reported cascade of events relating *FTO* upregulation to melanoma tumorigenesis is suggested to be initiated by metabolic stress, a metabolic challenge faced by tumor cells, which in turn induces *FTO* upregulation as a means of melanoma adaptation to metabolic challenges. Furthermore, *FTO* knockdown alleviated melanoma resistance to anti-PD-1 treatment; suggesting that FTO inhibition in combination with anti-PD-1 immunotherapy is a promising anti-melanoma therapy [103].

In line with the reported oncogenic role of FTO demethylase in a subset of AMLs [90] and the anti-tumor effect exerted by an FTO inhibitor (R-2HG) [92], Huang et al. [104] utilized structure-guided design to optimize Meclofenamic acid (MA), a previously identified FTO inhibitor [91]. This led to developing FB23 and FB23-2, as two more potent inhibitors of FTO-mediated demethylation [104]. The MA-derived FB23-2 FTO inhibitor was shown to inhibit proliferation and to induce differentiation of human AML cell lines, as well as to inhibit AML progression in-vivo using mice models. The inhibitory effects were mediated by upregulating *RARA* and *ASB2* (negative regulation) and downregulating *MYC* and *CEPA* (positive regulation), as a result of elevated m6A levels on transcripts following FTO inhibition (Figure 2) [104].

In endometrial carcinoma (EC), FTO overexpression promoted endometrial cancer proliferation and invasion, through PI3K/AKT and MAPK signaling pathways [82]. A recent study further pointed out that FTO overexpression in EC leads to m6A hypo-methylated *HOXB13*, a homeobox transcription factor. Hypo-methylated *HOXB13* transcripts are protected from YTHDF2-mediated decay, thus promoting higher HOXB13 expression and activating the Wnt signaling pathway. This in turn promotes EC invasion and metastasis [105].

Alterations to m6A modification play various roles in cancers, as the methylated target genes can be proto-oncogenes or tumor suppresser genes, as presented with FTO differential expression levels in pancreatic cancer [106,107]. Tang et al. [107] reported *FTO* overexpression in pancreatic cancer. They further indicated that *FTO* knockdown led to a compromised proliferation in pancreatic cancer cells, and in terms of its m6A demethylase activity, led to reduced *c-Myc* mRNA transcript levels by modulating its m6A enrichment [107]. In this study, *c-Myc* oncogene was investigated for its reported regulatory role in the cell cycle by mediating entry into S-phase [108]. While all studies discussed thus far highlight FTO’s oncogenic role in various cancers, FTO’s tumor suppressor function was recently uncovered by Zeng et al. They report reduced FTO expression in pancreatic cancers, which increased m6A levels on *PJA2* promoting its decay via YTHDF2. Suppression of PJA2 expression enhances Wnt signaling; thus promoting proliferation and invasion in pancreatic cancer cells [106]. These two studies on FTO’s role in pancreatic cancer cells present a dual role of FTO in pancreatic tumorigenesis that ought to be addressed, as it is reported as a tumor-promotor by one study [107] while its tumor-suppressor role is addressed in another one [106].

Interestingly, FTO’s tumor suppressor function was also reported in Ovarian Cancer Stem Cells (OCSCs) [109]. Huang et al. pointed to FTO downregulation in ovarian cancer cells and stem cells. Through transcriptome-wide RNA-seq and m6A mapping, two phosphodiesterase genes (*PDE4B* and *PDE1C*) with m6A hyper-methylated levels were identified. Subsequently, these stabilized m6A hyper-methylated transcripts promote the hydrolysis of the second messenger cAMP. Thus, downregulating cAMP and promoting tumorigenesis and self-renewal [109]. Interestingly, Huang et al. proposed the potential identification of hyper-methylated transcripts in ovarian cancer cells via IGF2BP1-3 as an RNA binding protein, which leads to increased transcript stability and gene expression levels [110] Overexpression of wild-type *FTO* was shown to otherwise inhibit ovarian cancer stemness and tumorigenesis, while mutant enzymatically-inactive forms failed to do so; highlighting the necessary role of FTO as m6A demethylase in ovarian cancer [109].

Overall, studies support FTO critical oncogenic roles in various cancer types as an m6A demethylase, given its abnormal expression and effect on many target genes. Figure 2 below presents a visual summary of studies on FTO demethylase activity, its overexpression in several human cancers, and the affected signaling pathways.

## 4. FTO Inhibition in Tackling FTO-Associated Disorders

The emergence of numerous GWA studies relating *FTO* SNPs with obesity/overweight across different populations motivated further research into investigating FTO physiological and pathological relevance; yet, the underlying molecular role of FTO in adipogenesis and cancer requires further research. A number of studies have reported the use of biochemical inhibitors of FTO, considering its pathological implications in obesity and cancers for instance.

Chen and team [111] identified the first potent inhibitor of FTO in 2012, the natural product Rhein. Rhein acts as a competitive inhibitor versus the nucleic acid substrate; thus, hindering FTO m6A demethylation activity. Rhein inhibitory activity of FTO was further affirmed on mRNA level using BE(2)-C cells (a clone of the SK-N-BE(2) neuroblastoma cell line), and illustrated significantly higher m6A levels [111]. Another study identified small molecules termed 2OG analogs as potent FTO inhibitors [112]. Their investigations were based on the fact that FTO demethylation activity is 2-oxoglutarate (2OG)-dependent [1]. In their study, they highlight the four most potent inhibitors, of which inhibitor 19 was previously reported in [111]. This study illustrated FTO demethylase activity using non-cellular in-vitro tests, whereby 3-methylthymidine (3meT) served as a test substrate. Further testing of FTO demethylating activity using other FTO substrates, such as m6A being the most abundant mRNA modification and the best substrate for FTO discovered yet under physiological conditions [2], would be required.

Another research study by Huang’s group [91] identified Meclofenamic acid (MA) as a selective inhibitor of FTO m6A demethylase activity over ALKBH5. MA as a nonsteroidal anti-inflammatory drug (NSAID) [113], was shown to inhibit FTO independent of 2OG or iron ions chelation. In-vitro tests revealed that MA increased m6A levels in HeLa cells mRNA via selectively inhibiting FTO, as affirmed from FTO overexpression and subsequent treatment with MA. Interestingly, the FTO-MA complex revealed a novel specific interaction called ‘a β-hairpin motif within nucleotide recognition lid (NRL)’, which explains the selective inhibition of FTO by MA [91].

Again, pertaining to the 2OG-dependent enzymatic activity of FTO demethylase [1], a new compound (molecule 7d) was developed by Zheng et al. [114] as an inhibitor of FTO enzymatic activity; inducing an increase in m6A total cellular RNA from HeLa cells. Their study presented 7d as the first FTO inhibitor with anticonvulsant activity, as affirmed from an animal model of pharmaco-resistant epilepsy (termed six Hz model) [114]; yet future investigations on this inhibitor effect on FTO activity in relation to obesity and cancers as FTO-associated disorders would be interesting. Toh and team [115] identified a selective inhibitor of FTO demethylase activity that illustrated increased m6A using a newly-synthesized compound 12. Interestingly, Glu234*_FTO_*residue was found to be a key determinant for FTO affinity and specificity towards its substrates; as other equivalent residues from AlkB subfamilies had weakened or abolished interaction with compound 12 [115].

Another newly synthesized compound N-(5-Chloro-2,4-dihydroxyphenyl)-1 phenylcyclobutane-carboxamide (abbreviated N-CDPCB, 1a) was reported [116], whose structure bound to FTO partially overlaps with MA [91]. He and the team affirmed N-CDPCB inhibitory activity of FTO catalytic action, which showed that m6A levels were increased in mRNA of *FTO*-overexpressing 3T3-L1 pre-adipocytes as compared to untreated cells. They also reported preliminary inhibitory activity of FTO demethylase activity on 3meT as an initial testing substrate using non-cellular assays [116]. A recent study re-visited the use of Rhein as a putative inhibitor of FTO [117]. In their investigation, Huang et al. provided mechanistic insights into the cellular actions by which Rhein modulates adipogenesis. Interestingly, by comparing Rhein-treated and *FTO*-knockdown groups, adipocyte formation was impaired in both groups. Nonetheless, Rhein was shown to have m6A-independent targets, including the novel Receptor Expressing-Enhancing Protein 3 (REEP3). Huang group reports that REEP3, an endoplasmic reticulum (ER) regulator, promotes adipogenesis in an m6A-independent manner and represents a druggable candidate target for obesity therapeutics [117]. The aforementioned biochemical inhibitors reported by different research groups [91,111,112,114,115,116] provide insightful findings regarding FTO inhibition, to better understand its potential physiological function and clinical value. Yet, none has been critically evaluated in terms of their in-vivo efficacy in the context of FTO pathological implications like obesity or cancers.

A study in 2019 by Peng and colleagues [73] repurposed entacapone, an FDA-approved drug for treating Parkinson’s disease [118], as a potent chemical inhibitor of FTO. Their mechanistic findings showed that *forkhead box protein O1* (*FOXO1*) mRNA is a downstream substrate of FTO, whereby entacapone inhibits FTO demethylase activity; resulting in m6A hypermethylated *FOXO1* mRNA and reduced FOXO1 expression levels. A novel FTO-FOXO1 regulatory axis was identified using a high-fat diet-induced obese (DIO) mouse model, in which FTO inhibition via entacapone modulated hepatic gluconeogenesis as observed from lower fasting blood glucose levels, as well as modulating thermogenesis in adipose tissue as observed from increased *UCP1* expression [73]. Nonetheless, the problematic high dosage used in animal studies and the differences between human and mice adipose tissue metabolism, necessitate the validation of entacapone use in humans and perhaps the development of entacapone analogs with better pharmacokinetic properties in the future.

A summary table of reported studies on biochemical inhibitors of FTO is illustrated in Table 2 below, highlighting current limitations as avenues for further future validations.

## 5. Concluding Remarks and Future Directions

Despite the discovery of RNA m6A in the 1970s [119,120] as the most prevalent modification found in eukaryotic mRNA; breakthroughs in better understanding its physiological implications began to be realized upon the identification of FTO as the first m6A demethylase of this highly dynamic and reversible process, as well as the development of m6A sequencing technologies. Dysfunctions in FTO m6A demethylase activity has been implicated in various human malignancies; highlighting m6A’s clinical role with insightful prognostic values for patients. In fact, given m6A regulatory role in various cancers, RNA methylation profiling can be utilized as a clinical tool in the future. The use of FTO inhibitors in conjunction with existing cancer therapies holds promising avenues for *FTO*-high expression tumors exhibiting resistance to cancer therapies as previously discussed [92,103]. FTO function in different cancers is context-dependent, whereby FTO may function as a tumor promoter or suppressor [106,107] by regulating various tissue-specific targets involved in unique pathways pertaining to different cancer types. Furthermore, taking into consideration that tumor angiogenesis is one of cancer microenvironment hallmarks [121] and that anti-angiogenic drug administration holds promise in improving treatment response [122]; further investigation on the involvement of m6A as an epitranscriptomic modification in cancer angiogenesis is needed. Current research on m6A involvement in cancer angiogenesis has mainly focused on an m6A writer METTL3 [123]; hence, future studies on other m6A regulators are required in the near future.

Several studies highlighted the underlying molecular mechanisms by which FTO is associated with obesity, given its role as an m6A demethylase, by denoting target gene transcripts that are affected by this post-transcriptional modification. Nevertheless, and as can be seen from Table 1, most adipogenesis studies have focused on *FTO* knockdown or overexpression in murine pre-adipocytes, which while advantageous does impose some limitations on adipogenesis studies. Since SNPs can impair function rather than complete loss of function (as performed in mice knockout studies); there is a need for more accurate models that reflect human *FTO* variants and their consequences in adipogenesis and obesity. Despite the benefits of mice 3T3-L1 pre-adipocytes in uncovering molecular mechanisms in adipogenesis; results obtained from human models are more reliable as they mimic human disorders, like obesity. In fact, primary human pre-adipocytes are utilized for the validation [124,125] or contradiction [124,126] of findings obtained using adipocyte animal models. Human primary pre-adipocytes are good tools for studying adipogenesis, and they can be derived from adipose tissue to produce in-vitro mature adipocytes under appropriate conditions. However, the drawbacks with these cells are their scarceness within fat tissue, and their limited renewal capacity [124]. Human-induced pluripotent stem cells (iPSCs) represent an attractive ideal cellular model that is promising in delineating mechanistic details of *FTO* SNPs on adipocyte differentiation, including production of thermogenic adipocytes at higher frequencies [127,128]. Several reports published directed differentiation protocols to produce thermogenic adipocytes using iPS cells without gene transfer methods [129,130,131,132]. Using iPS cells to produce thermogenic adipocytes and to investigate FTO’s role as an m6A demethylase throughout the adipogenesis process has not been investigated yet. This is a new and exciting area as it allows the temporal assessment of FTO and m6A during adipogenesis, including the earliest steps of adipogenesis such as adipocyte progenitors. This can bring about fundamental insights into adipogenesis regulation and the role of m6A in metabolic conditions like obesity.

Common pathways that are affected by FTO aberrant m6A regulatory functions in adipogenesis and tumorigenesis may likely exist, and ought to be further explored. Epidemiological studies reported strong associations of *FTO* SNPs with obesity, as well as various cancers. Some tumorigenic *FTO* SNPs were reported to be BMI-related [75,77,79] while others were independent of BMI [74,78,80,81]; indicating that obesity may confer increased risk in some cancers, through a potentially common FTO metabolic-tumorigenic regulatory axis. For instance, Liu and his team showed that *FTO* overexpression in breast cancer cells affects energy metabolism via the PI3K/AKT signaling pathway [86]. Autophagy might be a common regulatory mechanism in both disorders. Wang and colleagues report that FTO adipogenic regulatory role is exerted via ATG5 and ATG7-CEBPB signaling axis; delineating an underlying mechanism for the crosstalk between autophagy and adipogenesis [65]. A preliminary report by Zhao et al. pointed FTO’s oncogenic role in ovarian cancer, where FTO upregulation increased autophagy function of ovarian cancer cells, through increased autophagy-related 5 (ATG-5) protein levels [133]. Although this report is preliminary and lacks investigations on m6A levels, it points to the role of autophagy in tumorigenesis. Interestingly, the mTOR signaling pathway is a regulator of several cellular processes including autophagy, and is implicated in different disorders including cancer and obesity [134]. FTO-mediated tumorigenic role in endometrial cancer was uncovered and connected to the mTOR pathway [84,85,86], while mTOR’s connection to FTO-obesogenic functions awaits further exploration. In summary, molecular studies thus far are either focused on FTO molecular roles in adipogenesis or tumorigenesis, which will contribute to better understanding FTO molecular implications and awaits further investigations to decipher FTO’s potential dual function in obesity and cancer through common pathways.

Studies reviewed herein demonstrate that FTO is indeed an interesting druggable target in different human disorders, specifically cancers. Developing selective FTO inhibitors is still underway as studies investigating FTO inhibition have merely been tested in-silico context and some were tested in-vitro context. This necessitates the need for more in-vivo investigations as was performed in [73] which utilized a diet-induced obese (DIO) mouse model to investigate FTO inhibition in the context of obesity and adipogenesis. Performing in-vivo testing of FTO inhibitors is lagging; making it one of the current limitations in the field. Using relevant pre-clinical animal models can facilitate shifting these inhibitors into future clinical contexts. For example in the field of cancer therapeutics, patient-derived xenograft (PDX) models which were established by transplanting patient-specific tumor tissue into animals by surgery, were shown to maintain tumor microenvironment [135]. PDX models can be utilized as tools for personalized medicine strategies and cancer drug screening studies [135,136], such as testing FTO inhibitors in relation to its m6A-demethylase tumorigenic role.

As nearly one-third of mRNAs are m6A modified, the number of target genes regulated by FTO is relatively large. With such a large network of targeted transcripts reliant on the FTO enzymatic role, dysregulations in FTO m6A demethylase function must be further explored. Additional research into the biological implications of m6A modifications in various human pathologies, such as obesity and cancer, are required to fully understand their roles in such diseases and to develop precise therapies such as FTO small-molecule inhibitors in conjunction with traditional therapies, for better outcomes. Moving from GWAS signals to molecular and biological underpinnings necessitates the use of all technologies at our disposal, including third-generation sequencing technologies to highlight novel epitranscriptomic signatures in association with human disorders.

## Figures and Tables

**Table 1:** Summary of studies on FTO molecular mechanisms in relation to adipogenesis.

Finding	Year	FTO Manipulation	Cell Model Used	Reference
FTO regulates ghrelin m6A levels and subsequently mRNA abundance	2013	N/A *FTO* SNP rs9939609 (risk allele A)	Peripheral blood cells from AA subjects used in m6A methylation investigation	Karra et al. [52]
FTO deficiency was reported to upregulate uncoupling protein 1 (Ucp-1) and subsequently enhanced mitochondrial uncoupling and energy expenditure; resulting in induction of brown adipocyte phenotype	2013	*FTO* knockdown via short hairpin RNA (shRNA)	Human adipocytes and pre-adipocytes (from 3 healthy donors)	Tews et al. [59]
Inverse correlation during adipogenesis between m6A levels and FTO gene expression; the regulatory role of FTO in splicing of RUNX1T1	2014	*FTO* depletion via siRNA	3T3-L1 pre-adipocytes	Zhao et al. [10]
Negative regulation of lipid accumulation by m6A levels in porcine adipocytes	2015	*FTO* knockdown with shRNA	Porcine adipocytes	Wang et al. [49]
FTO adipogenic effect is mediated through mitotic clonal expansion (MCE); an early stage in adipogenesis	2015	*FTO* knockdown via siRNA	Primary adipocytes and MEFs from genetically modified mice models	Merkestein et al. [46]
FTO functional requirement for pre-adipocyte differentiation	2015	*FTO* knockdown via siRNA	3T3-L1 pre-adipocytes	Zhang et al. [54]
*FTO* SNP rs1421085 results in an increase in energy-storing white adipocytes, reduced mitochondrial thermogenesis, and increased lipid storage	2015	*FTO* SNP rs1421085 (T-to-C)/causality further investigated using CRISPR/Cas9 genome editing	Human adipocyte progenitor cells	Claussnitzer et al. [60]
Novel regulatory mechanism of FTO as m6A demethylase in regulating lipid accumulation in skeletal muscle cells	2017	*FTO* knockdown via siRNA	Mouse myoblast cell-line C2C12; Wild-type and obese mice models used for muscle collection and analysis	Wu et al. [61]
Epigallocatechin gallate (ECGC) shown to inhibit adipogenesis by inhibiting the MCE stage through targeting FTO as an m6A demethylase and in an m6A-YTHDF2-dependent manner	2018	*FTO* reduced via: an inhibitor (ECGC) siRNA	3T3-L1 pre-adipocytes	Wu et al. [62]
Zinc Finger Protein 217 (Zfp217) reported as a regulator of adipogenesis by activating FTO m6A demethylase at the transcriptional level, and by interacting with YTHDF2 post-transcriptionally	2019	N/A *Zfp217* knockdown via siRNA	3T3-L1 pre-adipocytes	Song et al. [65]
Entacapone identified as an inhibitor of FTO by mediating metabolic regulation through FOXO1 (as a direct substrate of FTO)	2019	An inhibitor (entacapone) shRNA	Diet-induced obese (*DIO*) mouse model, diabetic db/db mice; and Hep-G2 hepatic cells (for *FTO* knockdown by shRNA)	Peng et al. [73]
FTO as an m6A demethylase reported as a regulator of autophagy and adipogenesis	2019	*FTO* knockdown via siRNA	3T3-L1 pre-adipocytes; porcine primary adipocytes and *FTO-KO* mice	Wang et al. [71]
A link between metabolism and RNA m6A methylation was illustrated, where NADP binds FTO and enhances its activity; thus promoting m6A demethylation and adipogenesis	2020	*FTO* knockdown via siRNA	3T3-L1 pre-adipocytes and *FTO-KO* mice	Wang et al. [70]
FTO deficiency promotes thermogenesis and white-to-beige adipocyte transition via YTHDC2-mediated translation and enhanced protein expression of HIF1A	2021	Adipose-specific *FTO* knockout mice (*FTO*^AKO^)	*FTO* knockout mice (*FTO*^AKO^) and 3T3-L1 pre-adipocytes	Wu et al. [72]

**Table 2:** Reported biochemical inhibitors of FTO enzymatic activity.

Inhibitor	Outcome(s)	Limitation(s)	m6A Evaluation Assay	Reference
Rhein	Inhibitory activity verified using in-silico, biochemical and cellular tests	- In-vivo specificity remains to be explored to further evaluate efficacy	Liquid chromatography–mass spectrometry assay (LC-MS/MS)	[111]
	Modulation of RNA methylation and adipogenic differentiation by Rhein demonstrated to occur in a separate manner	- In-vivo specificity remains to be explored to further evaluate efficacy - In-vitro tests performed using murine pre-adipocytes only (validation using human primary adipocytes is needed)	Methylated RNA immunoprecipitation sequencing (MeRIP-seq),	[117]
2OG analogs	Biochemical and crystallographic tests were used to illustrate the inhibitory activity of four potent inhibitors	- No testing of inhibitory activity using in-vitro cellular assays nor in-vivo testing. - Inhibitory activity verified using demethylation tests on 3-methylthymine only, hence, other FTO substrates ought to be tested.	Liquid chromatography (LC)	[112]
Meclofenamic acid (MA)	Biochemical, crystallographic, and in-vitro (HeLa cells) tests confirmed binding and inhibitory activity	- In-vivo specificity remains to be explored to further evaluate efficacy	High-performance liquid chromatography (HPLC)-based assay	[91]
Newly synthesized compound (7d: N-(3,4-Dihydroxy-5-(4-chlorophenyl)-2-furanyl)- Ethanesulfonamide)	Computational docking to confirm binding activity, and in-vitro testing (HeLa cells) to evaluate cellular m6A levels	- In-vivo specificity remains to be explored to further evaluate efficacy (in context of m6A levels) - Animal models used focused on epilepsy as per study purpose; other FTO-related disorders ought to be investigated (e.g., obesity and cancer)	LC-MS/MS	[114]
New synthesized compound: (compound 12: 4-[N’-(4-Benzyl-pyridine-3-carbonyl)-hydrazino]-4-oxo-but-2-enoic acid)	Crystallographic, molecular modeling, biochemical and in-vitro (HeLa cells) testing confirmed binding and inhibitory activity	- In-vivo specificity remains to be explored to further evaluate efficacy	HPLC-based assay	[115]
N-CDPCB (1a)	Crystallographic, molecular modeling, biochemical and in-vitro (3T3-L1 pre-adipocytes) testing confirmed binding and inhibitory activity	- In-vitro tests performed using murine pre-adipocytes only (validation using human primary adipocytes is needed) - In-vivo specificity remains to be explored to further evaluate efficacy	LC-MS/MS	[116]
Entacapone	Inhibitory activity of this repurposed drug was affirmed after virtual screening and in-silico testing using in-vitro (hepatic Hep-G2 cells) and in-vivo (DIO mice) assays	- High drug dosage utilized in animal models; inability to extrapolate results from conducted mice studies directly to humans	M6A-antibody pulldown assay combined with m6A RNA seq; helped in evaluating m6A levels as well as identifying a substrate using transcriptome profiles	[73]
